# “Challenges and concerns faced by parents of a group of Egyptian children with cleft lip/palate: a qualitative study”

**DOI:** 10.1186/s12903-023-03747-9

**Published:** 2023-12-16

**Authors:** Ahmed Osama Morsi, Alaa Mohammed Yehia, Amira Saad Badran, Nagwa Mohammed Ali Khattab

**Affiliations:** Faculty of Dentistry, Ain Shams University, Cairo, Egypt

**Keywords:** Cleft lip/ palate, Qualitative study, Parental emotions, Challenges, Concerns, Parental attitudes

## Abstract

**Background:**

Cleft lip and palate are the most common developmental anomalies that affect the mouth and related structures. They can both affect children physiologically, socially, and functionally and lead to psychological distress in their parents. The present study aims to understand the challenges parents of cleft lip and palate patients face in Egypt, elucidate how they cope with these challenges, and assess their concerns for the future.

**Methods:**

For the present phenomenological qualitative exploration, the parents of cleft lip and palate patients attending the cleft care clinic were invited to participate in the study through face-to-face recruitment at the clinic. An interview guide about the research question was developed to include standardized open-ended questions providing a framework for structured discussions. The interviews were audio-recorded after obtaining written informed consent from participants then collected data were transcribed for data analysis.

**Results:**

Of the 12 participants, there were nine mothers and three fathers. Their children’s ages ranged from 1.5 years to 19 years and had different presentations of cleft lip and palate from unilateral cleft lip to complete bilateral cleft lip and palate. Feeding difficulty was one of the main challenges encountered by the parents. At the same time, fear of being subjected to bullying was the main concern for the future of their children. Six themes were noted that were continually reported: Health & Wellbeing; Parental emotions; Parental attitudes & behaviors; Financial aspects; Relationship aspects; and Career/Education.

**Conclusions:**

There were 4 factors that directly impacted the themes, namely: the type of cleft, gender of the child, gender role of the parent, and the age of the child impacted the parental concerns and the challenges faced under the influence of sociocultural beliefs and existing support systems.

## Background

Cleft lip and palate (CLP) are the most common congenital anomalies that affect the mouth and related structures separately or syndromically. A systematic review in 2021 revealed that the global prevalence of CLP is 0.45 in every 1000 live births, with wide variation in different studies and populations [1]. In Egypt, a study conducted in 2019 showed that the prevalence of oral clefts is 4 per 10,000 births and that cleft lip (CL) occurs more often than other types of oral clefts and its main cause is maternal passive smoking [2].

The clinical manifestations of CLP vary in terms of position/location, extent, and severity. It can be unilateral or bilateral, ranging from a notch over the lip to a complete cleft of the lip and palate. These clefts are multifactorial, influenced by genetic factors and exogenous factors, such as maternal malnutrition, hormonal disorders, tobacco consumption and medications during pregnancy, and other biological factors [3]. Prenatal identification of CLP by ultrasonography helps to provide time for parental education about its potential causes, how to deal with the child after birth, and procedures that the child may need later [4].

Research studies have shown that CLP is associated with physiological, social, and functional problems in affected children and psychological distress in both children and parents [5, 6]. Children born with CLP have far greater challenges in achieving and maintaining optimal oral health and a satisfying quality of life, which has a significant impact on their overall health and happiness as well [7].

Patients with CLP need to be treated at the right time and at the right age to achieve functional and aesthetic well-being. The treatment process is a complex multidisciplinary approach that is best provided by an interdisciplinary team of oral and maxillofacial surgeons, orthodontists, pediatric dentists, dental public health professionals, plastic surgeons, prosthodontists, psychologists, nurses, and speech therapists [8]. It requires multiple interventions including surgical and rehabilitation procedures, and frequent follow-up appointments from birth into adulthood to address problems related to impaired facial growth, speech impairment, hearing difficulties, and dental anomalies [9]. As such, CLP can bring a range of additional life stressors, affecting emotional, social, and financial resources and the well-being of the family unit; particularly parents [5]. CLP is a major dental public health concern, and even though not directly involved in the clinical treatment, dental public health practitioners have a crucial role in a larger context. They play a role in understanding how the anomaly develops, its causative factors and its impact on health and wellbeing, as well as partake in spreading awareness.

Previous studies found that parents of newborn babies with CLP needed information and support in feeding [9, 10]. A systematic review revealed that several studies were conducted to assess parents’ perceptions about the cause of CLP, the coping/adaptation strategies to such a condition, mother-infant interaction/attachment, the quality of life of their children, and outcomes of care services [11]. Another study evaluated the impact of having a child with CLP on the parents’ quality of life [12]. However, to the best of our knowledge, there is insufficient information regarding the experiences and challenges faced by the parents of CLP children in Egypt. Phenomenological qualitative research is one of the best methods to borrow the lived experiences of participants to better understand a specific phenomenon, and to understand the perspectives of the participants by exploring the psycho-social aspects of care [13, 14]. Hence, the present study aims to understand the challenges parents of CLP patients face in Egypt, elucidate how they cope with these challenges, and assess their concerns for the future.

## Participants and methods

For the present phenomenological qualitative exploration, data about the challenges faced by parents of children with CLP and their concerns for the future were collected at the cleft care clinic, Faculty of Dentistry, Ain Shams University. Ethical approval was sought from the Research Ethical Committee, Faculty of Dentistry, Ain Shams University (Reference number: FDASU-Rec IR052203). The present study was conducted in accordance with the guidelines of the Declaration of Helsinki. The present study was registered at ClinicalTrials.gov, with registration ID: NCT05730946.

### Participants

The parents of cleft lip and/or palate patients attending the cleft care clinic for the treatment or follow-up of their children were invited to participate in the study through face-to-face recruitment at the clinic. This centre treats CLP patients from all over Egypt as it is one of the biggest and most famous CLP centres in Egypt. Only nonsyndromic CLP patients were included in the study. Based on who accompanies the child to the clinic, either mothers or fathers were invited to participate in the study to explore their different perceptions about the challenges and concerns. CLP patients with current pain and those who needed emergency treatment were excluded so that their parents’ perceptions were not affected. Participants were informed about the goals of the study and assured of data confidentiality. Written informed consent was obtained from parents regarding their participation and the recording of the interviews.

In qualitative studies, guidelines for sample size calculation are based on the concept of saturation rather than sample size estimation. In the present study, the saturation strategy used is the stopping criterion approach where saturation is assumed when 2 consecutive interviews do not provide new information or insights [15]. Saturation was first noted in the 9th interview; however, interviews continued to 12 interviews to ensure saturation. This number of interviews is acceptable and aligns with findings reported by Hennink et al. [15] that most datasets reached saturation between 9 and 17 interviews, with a mean of 12–13 interviews.

### Data collection

An interview guide about the research question was developed by the authors to include standardized open-ended questions in which participants were asked almost the same questions [16]. It provided a framework for structured discussions and stimulation of the interaction between the researcher and the participants. However, participants could also raise issues outside the framework that they considered to be important.

The methodical structure of the interviews was chronologically and consistently arranged including (a) introduction and warm-up questions, such as how did you feel when you knew you were pregnant, how did you tell your family, and how did the child’s father/mother (according to who was asked the question) receive this news?, (b) transition questions such as what is the best thing about children, and what is the most difficult thing about having a child in general? (c) core questions, such as how did you feel when you found out that your child had cleft lip/palate, what were the challenges you faced as a mother/father of a child with a cleft lip/palate, and what are your possible concerns about your child’s personal life in the future? and (d) wrap-up and ending questions such as what did you want to know about cleft lip/palate before giving birth, what would you like to tell the doctors about your experience, and would you like to tell us anything else? [17]. The main topics included in the interview guide were the challenges of raising children generally, the experience of learning about the CLP condition of the child, the unique challenges of being a parent of a child with CLP, and the concerns for the future regarding the child’s health. The participants were also asked to provide some keys for the healthcare providers about how to deal with CL/P patients and their parents.

It was piloted in two interviews to assess how the participants understood and responded to questions. No modifications were made to the interview guide. Data were collected using the interview guide during the year 2022 from July to November. All interviews were moderated by the same researchers in the same facilities to ensure consistency in the processes. The interviews were scheduled on the same days as the participants’ cleft care clinic appointments. The interviews were audio-recorded, and the collected data were transcribed for data analysis.

### Data analysis

The audio recordings from the interviews were transcribed verbatim in Arabic by one author to ensure standardization of the transcription process. The transcripts were reviewed by two authors (AM, AO) independently and thematic content analysis was performed in which the main themes and subthemes were extracted from the data [18]. Meaningful “text units” were extracted manually through line-by-line coding. Thematic interrelation was discussed between the two authors to attain an agreement. Ultimately, the refined final version of the codes was applied to the total transcriptions independently one more time, and agreement was attained between the two coders. Quotations were selected to illustrate the observed themes and subthemes. The items/statements were then derived by the authors from the selected quotations. All quotations included in the paper were translated into English by the authors, and the transcripts and interviews were conducted in Arabic, particularly the Egyptian dialect. The symbols used in transcripts are included in Table 1.

**Table 1:** Symbols used in transcripts

Punctuation mark / symbol	Indication
….	Indicating a prolonged pause or a sudden change of sentence
( )	Indicating a word that we added to the sentence to better convey the participant’s point
“ ”	Indicating a direct quotation made by the participant
[ ]	Indicating translation made by the authors.

The authors analyzed the data using a thematic content analysis approach. According to Green & Thorogood [19], this approach aims to “provide a map of the contents and topics” across the dataset and summarize the “variations and regularities within the data”. After analyzing each interview, we held debriefing sessions to validate the codes and the coding process. For instance, we identified topics that needed to be explored deeper in future interviews and refined the coding process at every step of the analysis.

## Results

**Fig. 1:**
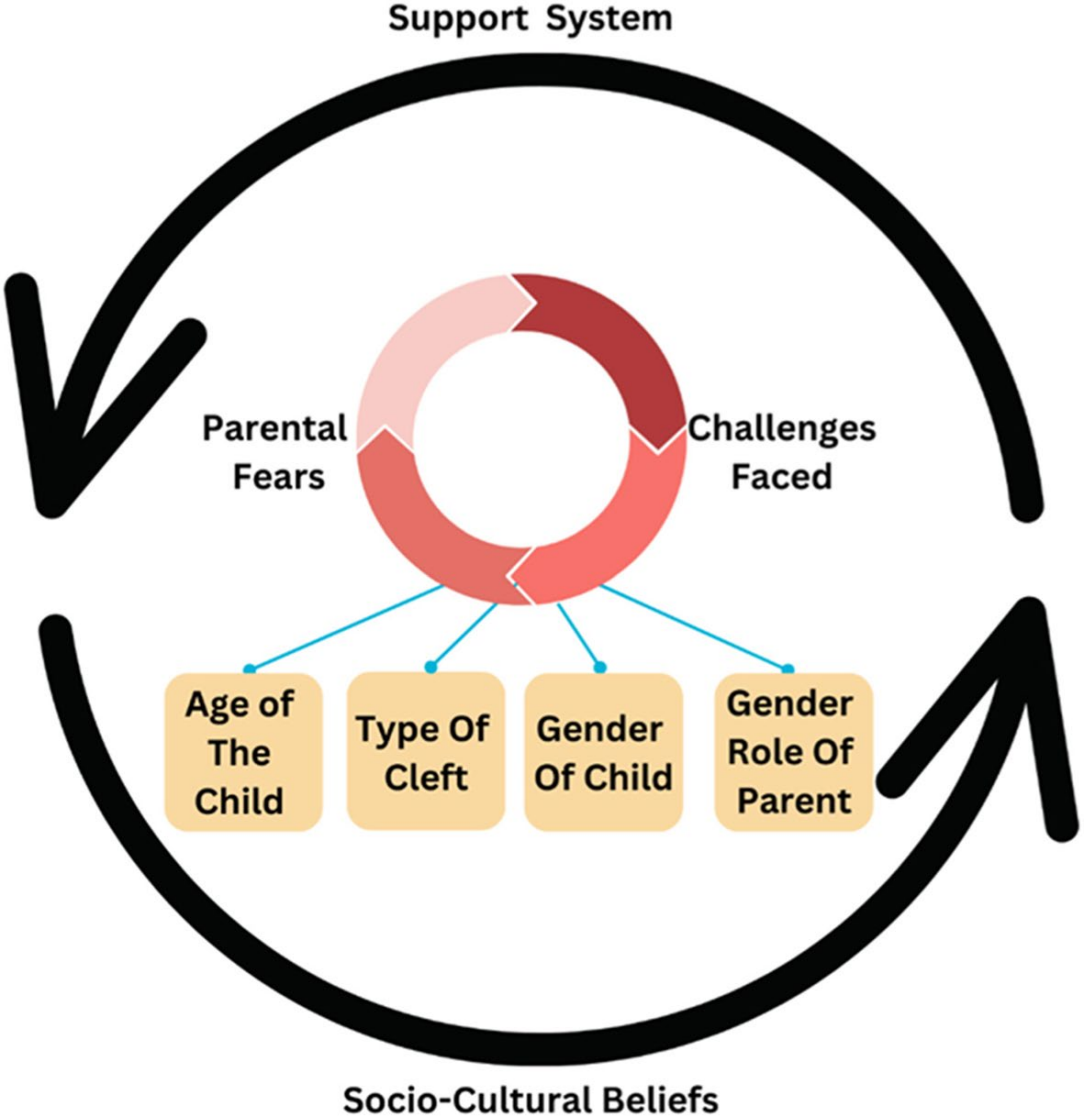
Theoretical framework

Of the 12 participants, there were nine mothers and three fathers. Their children had ages that ranged from 1.5 years to 19 years and had different presentations of CLP from unilateral cleft lip to complete bilateral cleft lip and palate. Saturation was noted in the 9th interview, and the 10th through 12th interviews were performed to confirm saturation. There were six themes noted that were continually reported, namely: Health & Wellbeing; Parental emotions; Parental attitudes & behaviors; Financial aspects; Relationship aspects; and Career/Education. As shown in Fig. 1, there were 3 factors that directly impacted the themes, namely: The Type of cleft, Gender of the Child, and the Gender Role of the Parent impacted the parental concerns & the challenges faced under the influence of sociocultural beliefs and the existing support systems. For the sake of data protection, pseudonyms were given to all participants as shown in Table 2. It is important to note that for each child, only one parent was included.

**Table 2:** Demographic characteristics of the participants

Pseudonym	Relationship to the child	Child’s age (in years)	Child’s sex	Cleft lip and/or palate type
**Rania**	Mother	4	Female	Unilateral CL
**Yasmin**	Mother	8	Male	Unilateral CLP
**Dina**	Mother	19	Female	Unilateral CLP
**Mostafa**	Father	14	Male	Unilateral CLP
**Mona**	Mother	2.5	Male	Unilateral CLP
**Salma**	Mother (Widowed)	12	Female	Unilateral CL
**Karim**	Father	15	Female	Unilateral CLP
**Aya**	Mother	17	Male	Bilateral CLP
**Mohamed**	Father	1.5	Male	Bilateral CLP
**Mariam**	Mother	14	Female	Unilateral CLP
**Ganna**	Mother	12	Male	Unilateral CL
**Menna**	Mother	3	Male	Unilateral CLP

### Theme 1: parental emotions

Parental emotions ranged from acceptance to fear & disgust, and there was a great variation of experiences across the span of the child’s lives.

In the first few hours, fathers expressed fear, anxiety and an overall sense of incompetence experienced during the first hours after the delivery of the child, as they were tasked by nurses/medical personnel to feed and take care of the child, as well as find appropriate hospitalization for them:

[It felt like a truck ran over my head, I couldn’t think.]

Mothers expressed varying emotions, with one participant expressing an overwhelming sense of fear and disgust:

[I was taken aback and worried. My reaction was very aggressive when I saw her. It was the first time I ever saw a baby looking like that, did not even know that was possible. I was taken aback and scared.]

And another mother, Aya, experiencing absolute acceptance:

[I was totally ok, I don’t know how, I usually cry at the slightest thing. However, that day, I was calm and collected for some reason. God bestowed acceptance upon my heart, accepted it then, accepting it now.]

While Salma reported a sense of guilt:

[The feeling then was of guilt, maybe I am the reason that happened, maybe I was not taking care of my health during the pregnancy.]

It was worth noting however, a link between religiosity and acceptance was noted as shown below when Yasmin said:

[The first thing I did was to show gratitude to God, and prayed for help in raising him…].

And Menna said:

[His father was also very happy, because when we had Mahmoud – we said we would name our son Mahmoud if we ever had one- so when we had him, he brought joy into our lives. We had him in difficult times, but thanks to God everything went smoothly.]

### Theme 2: parental attitudes & Behaviours

Parental attitudes and behaviours were highly correlated, so they were considered one theme. Parents with supportive attitudes showed behaviours that support the child; for instance, when Karim was asked about any future worries, he replied:

[Well there is nothing, the future is in God’s hand, all I do is support her as much as possible.]

On the other hand, parents who did not consider CLP as “normal” displayed behaviours to overcompensate, such as contrasting the child against others for a sense of normalcy as Salma did:

[So for instance her sister has everything normal, but her hair is truly bad. Therefore, I always tell her, look at your sister, her hair is truly bad.]

### Theme 3: Health & Wellbeing

This theme originally focused on behaviour by medical personnel, but after further analysis it was noted that behaviour by medical personnel is but a subtheme of the Health & Wellbeing theme. The health and wellbeing of the child was noted as a core theme that was affected by multiple subthemes. Behaviour by medical personnel greatly impacted the willingness of parents to follow-up with the doctor. Mothers were more likely to note how empathetic and responsive the Dr. was.

[So for how I like them to behave, I want them to show care, so when I call them, they answer. I text them via WhatsApp they reply ASAP, especially when he has just done a surgery or during surgeries.]

Fathers were more likely to focus on how “professional” the Dr. was, which was judged by cleanliness and being orderly.

[First things first, is, erm, how the Dr. Meets the patient, erm, I mean his punctuality.]

A major challenge primarily reported by fathers, was the access to healthcare services, particularly in the first few hours after delivery:

[She told me he has a hare lip and a cleft palate and that they can’t feed him, and that I had an hour to deal with it. Look for a public hospital that can manage this.]

Another key component of healthcare was related to breastfeeding and formula feeding, where almost all participant mothers reported it as a challenge. Salma, who was breastfeeding, explained how it was emotionally taxing and how it was physically difficult.

[Of course, at first, I cried my eyes out. It was very difficult; breastfeeding was almost impossible. Till, by some divine miracle, she got used to it and she knew how to.]

And Mariam, who was formula feeding, faced difficulty as her child almost always regurgitated:

[She was very weak when she was first born. Breastfeeding was difficult because she was bringing it (milk) from her nose. Oh, breastfeeding itself did not benefit her. She could not breastfeed naturally. Breastfeeding was challenging; everything was coming out from her nose.]

### Theme 4: financial aspects

Financial aspects were mentioned both as an actual challenge, where Karim explained that with prices rising, financial challenges have been a great stressor.

[Excuse me, but the cost in other places is very high. In addition, seriously it is stressful.]

And as a concern, where Mostafa clearly stated that he is anxious about the potential costs for future procedures and whether he can afford them.

[It (the concern) is cost related, Now the cost for plastic surgery is very high and it has become a trade.]

The financial aspects were not usually mentioned by mothers; however, they were constantly mentioned by fathers.

[It (the cost) is good around here; you know if I am asked for a sum of money, but I will see an improvement. It is just coz, why do we even make money, it is so my kids can live in better standards and have a decent life. SO that I treat them, and they are fine, way better than collecting money and seeing them sick, what would I benefit then?]

However, they were clearly mentioned by one mother, who was a widower and thus had to perform both gender roles.

[Additionally, the finances matter a lot, the prices in other places are astronomical, here it is completely different.]

### Theme 5: relationship aspects

This theme was complex and displayed a high variability. Relationships with peers were the most commonly mentioned. One common view was regarding bullying, which was viewed as a detriment to healthy peer relationships. For instance, Mohamed was worried that bullying would make his son’s behavior aggressive.

[This bullying might make him aggressive.]

Yasmin mentioned how it is vital for parents to know how to handle bullying.

[You need to know how to protect them from kids who bully them, for the sake of their mental health.]

The effects of bullying on both children and parents were reported, in some form or another.

Another subtheme was for romantic relationships, where it was reported by Mariam as a challenge as her daughter was rejected.

[Of course, if a suitor sees her, they would be scared that their kids might end up the same and would reject her, and it happened already.]

It was also reported as a concern. Concern for relationship status was more likely to be noted by mothers, particularly for girls:

[However, as a mom, I want my girl in the best picture, especially since she is a girl. I keep thinking about when a suitor comes and sees her…].

### Theme 6: career/education

Both career and education were considered as one theme since they went in line. Regarding education, all parents clearly stated that their children were “smart” and/or “intelligent”. Religiosity & acceptance played a component here, where some stated their children’s success in academics as a way for God to compensate them for their CLP; for example, Mostafa stated that his son’s intelligence is something that makes up for his condition.

[Very much so, he is funny and social, makes a lot of friends and is very smart. God compensated him with how smart he is.]

There were no challenges clearly stated in academics, however there was a concern reported by a parent regarding phonetics and how it can impact the child’s ability to learn.

Concern for education and employment was more likely to be noted for boys, especially by fathers.

[So what do you think of a child like that, his other brothers talk whether in fifth grade or sixth, but what about him, where/how will he end up?]

### Factor 1: type of cleft

The type of cleft greatly influenced the challenges faced by the parents and their concerns. For instance, parents of children with a unilateral CL did not face difficulties with lactation, as reported by Salma:

[Because she only had a cleft lip it did not affect breastfeeding, I could breastfeed naturally.]

However, those same parents greatly emphasized the aesthetic aspect as the major concern.

[I want to do another cosmetic surgery for her] because I feel there is still something…As a mother I want her in her best form, especially since she is a girl…I keep wondering what will happen when someone comes to propose to her.]

On the other hand, parents of children with bilateral CLP experienced greater difficulty with lactation, as stated by Dina:

[It (lactation) was difficult, particularly around the palate surgery].

And even faced the challenge of the child undergoing more surgeries:

[They (other parents) had told me that this was a more difficult surgery. In addition, it opened up and here I am, redoing it.]

Even though they were concerned about aesthetics, it was not their major concern.

### Factor 2: gender of the child

As discussed before, the gender of the child influenced how parents perceived the challenges, where parents of girls perceived aesthetics as a major concern and was linked as a hinderance to potential relationships. On the other hand, parents of boys were less likely to be concerned about aesthetics and were more concerned about careers.

### Factor 3: gender role of the parent

The gender role of the parent showed an interesting view on the challenges, in different aspects. As explained above, mothers cared more about empathetic medical personnel, while fathers cared more for punctual and “professional” medical personnel.

This was also shown in the financial aspect, where fathers were more likely than mothers to note the financial component as a heavy challenge and concern, the gender role was at full play here where a widowed mother also expressed the same level of concern when it came to the financial component.

### Factor 4: age of the child

As opposed to other factors, the age of the child was a factor that affected whether a certain theme was perceived as a concern or an actual challenge that was met.

## Discussion

The present study examined the experiences of parents of CLP patients to better elucidate their needs and develop more patient- and carer-centric approaches. Overall, the findings showcase the complexity of the parental experiences.

Participants in this study included both fathers and mothers, who were parents to both young and adolescents, boys, and girls. The study aimed at maximum variation to fully explore the experiences of the parents through the life journey of their children. Surprisingly, the core themes were very clearly apparent regardless of the variability; however, the impact of these themes on the parental experience was richly diverse, albeit not individually unique. It is important to note that the authors chose maximum variability sampling to attempt and capture the whole experience, and interestingly the age of the child affected how a certain theme moved from being a concern to a challenge. For example, for very young children, bullying was a concern (as they have yet to meet peers), but school aged children, bullying was a challenge as opposed to a concern.

Parental emotions were diverse, with shock being particularly apparent. The emotions differed throughout the life journey of the child and were clearly impacted by the existing support system. Fathers in particular, were shocked heavily, especially when asked by nurses to feed the child (as the nurses could not) and to find an appropriate hospital for the child. This finding is consistent with the results of previous studies which showed that feeding was one of the main concerns of the parents [20, 21]. This also clearly showcases the importance of caretaker counselling training for all healthcare professionals involved in the cleft lip and palate care team, and of particular importance the nursing staff.

Parental attitudes and behaviours were in turn influenced by their emotions. Some showed acts of overcompensation due to a sense of guilt. Another reported behaviour included social withdrawal. The findings of parental emotions, behaviours and attitudes are in line with previous research [22, 23].

Financial aspects, relationship aspects, and career & education presented as both an existing challenge and a rising concern. They were heavily influenced by the gender of the child and the gender role of the parent. Fathers were more likely to worry about career and financial aspects, while mothers were more concerned with relationship aspects, particularly for females.

These findings are only operational within the framework supported by sociocultural norms and existing support networks. This is clearly highlighted when viewing the effect of religiosity, as a core component of socio-cultural beliefs, on parental emotions and behaviours. Where religious beliefs impact parental understanding of the cause of the phenomenon and acceptance. The idea that sociocultural aspects of health have an impact larger than that of individual variation and behaviours is in line with existing developments in understanding social determinants of health [24–26].

The study findings are in line with existing literature, showcasing the impact of different factors on parental experiences. Parental emotions when receiving the news of CLP was highlighted as with previous research, an experience that was labelled as traumatic by Johansson and Ringsberg [9]. These findings also reiterate the importance of taking into consideration the psychosocial wellbeing of parents of children with CLP as well as enabling healthy coping strategies as was covered in previous research [5, 27, 28].

From these findings the following recommendations can be made:

- The establishment of a helpline to help direct healthcare providers and parents toward the nearest healthcare facility specialized in the management of congenital malformations. This will help tackle the challenge reported by parents in access to care and in finding proper healthcare services for their children.
- Specialized training in patient-centered care and caretaker counselling should be provided to healthcare personnel working with CLP patients. This will tackle the behavior by healthcare providers component.
- Create support groups for parents of children with congenital malformations. This will empower the social support framework and allow healthier management of parental emotions while encouraging positive attitudes and behaviours.
- Encourage obstetricians/gynecologists (Ob/Gyn) and other relevant clinicians to detect orofacial anomalies during pregnancy via a combination of 2D and 3D sonography [29].

### Study strengths and limitations

The use of qualitative methodology is the main strength of the present study as it allowed for an in-depth exploration of the challenges parents of CLP patients face in Egypt and their concerns for the future. Implications from this project aim to provide better holistic healthcare services for CLP patients and their families. However, limitations should be acknowledged. This study is based on a sample from one setting (cleft care clinic) due to limited accessibility to other hospitals treating CLP patients. Nevertheless, the centre is one of the biggest and most famous CLP centres in Egypt that treats patients from all over Egypt, furthermore, common themes were evident, and thematic saturation was reached. Future research is also needed on the experiences of the nursing staff dealing with CLP patients and their perceived training needs.

Finally, as this is a qualitative study, it inherently aims at exploring new themes and not generalizability of theories, thus a need for a follow-up quantitative study to assess the generalizability of the current study would be valuable, and it would also enable inclusion of factors not included during the data collection, but seemed valuable in retrospect, such as the order of the child and the socioeconomic standard of the parents.

## Conclusions

This qualitative study shows that even though the experiences varied widely at birth, they culminated in similar findings along the way. When exploring the experiences of parents of children with CLP, different themes emerge, namely, behaviour by medical personnel; parental emotions; Parental attitudes and behaviours; financial aspects; relationship aspects; and career/education. There were 4 factors that directly impacted the themes, namely: The Type of cleft; Gender of the Child; the Gender Role of the Parent and the Age of the Child impacted the parental concerns & the challenges faced under the influence of sociocultural beliefs and the existing support systems.

## Data Availability

The datasets generated and/or analyzed during the current study are not publicly available due to confidentiality but are available from the corresponding author upon reasonable request. All authors had full access to all data in the study and take responsibility for the integrity of the data.

## Abbreviations

CLP: Cleft lip and/or palate
CL: Cleft lip
Ob/Gyn: Obstetricians/Gynecologists

## References

[1] Salari N, Darvishi N, Heydari M, Bokaee S, Darvishi F, Mohammadi M (2021). Global prevalence of cleft palate, cleft lip and cleft palate and lip: a comprehensive systematic review and meta-analysis. J Stomatol Oral Maxillofac Surg..

[2] Rezq Alswairki HJ, Abd El-Sayed FA, Fouda MY, Fahim FH, Haque S, Alam MK (2019). Incidence of Egyptian live births of cleft lip and/or palate in Cairo, Luxor, Aswan and New Valley governorates: a survey study in 237,783 children. Pesqui Bras Odontopediatria Clin Integr..

[3] Pavlova NI, Kurtanov KA, Diakonova AT, Mironova LS, Solovyeva NA, Borisova YP (2020). Genetic predictors for the development of congenital orofacial clefts. Int J Biomed..

[4] Mednick L, Snyder J, Schook C, Blood EA, Brown SE, Weatherley-White RCA (2013). Causal attributions of cleft lip and palate across cultures. Cleft Palate-Craniofacial J..

[5] Baker SR, Owens J, Stern M, Willmot D (2009). Coping strategies and social support in the family impact of cleft lip and palate and parents’ adjustment and psychological distress. Cleft Palate-Craniofacial J..

[6] Srivastav S, Duggal I, Duggal R, Tewari N, Chaudhari PK, Pandey RM (2021). Parental response to the feeding behavior problems in children with cleft lip and palate: a systematic review. Spec Care Dent..

[7] Sahoo AR, Dheer SS, Mahesh PC, Goyal P, Sidhu R. A questionnaire study to assess patients with cleft lip and palate for their Oral health-related quality of life. Cureus. 2023;15:e38712. 10.7759/cureus.38712 PMC10246514 37292523

[8] American Cleft Palate-Craniofacial Association. Parameters for evaluation and treatment of patients with cleft lip/palate or other craniofacial differences. Cleft Palate Craniofacial J. 2018;55:137–56. 10.1177/1055665617739564 34162066

[9] Johansson B, Ringsberg KC (2004). Parents’ experiences of having a child with cleft lip and palate. J Adv Nurs..

[10] Owens J (2008). Parents’ experiences of feeding a baby with cleft lip and palate. Br J Midwifery..

[11] Nelson P, Glenny A-M, Kirk S, Caress A-L (2011). Child : parents ’ experiences of caring for a child with a cleft lip and / or palate : a review of the literature. Child Care Health Dev..

[12] De Cuyper E, Dochy F, De Leenheer E, Van Hoecke H (2019). The impact of cleft lip and/or palate on parental quality of life: a pilot study. Int J Pediatr Otorhinolaryngol..

[13] Ghirotto L, De Panfilis L, Di Leo S (2020). Health professionals learning qualitative research in their workplace: a focused ethnography. BMC Med Educ..

[14] Baker C, Wuest J, Stern PN. Method slurring: the grounded theory/phenomenology example, J Adv Nurs. 1992;17:1355–60. 10.1111/j.1365-2648.1992.tb01859.x 1430643

[15] Hennink M, Kaiser BN (2022). Sample sizes for saturation in qualitative research: a systematic review of empirical tests. Soc Sci Med..

[16] Turner DW (2010). Qualitative interview design: a practical guide for novice investigators. Qual Rep..

[17] Swartling AG. Focus group (FG). Adv Tools Sustain Assessment, Eur Comm Webb. 2007. p. 1–14.

[18] Anderson R. Thematic content analysis (TCA). In: Descriptive presentation of qualitative data, Vol. 15. 2007. p. 1–4.

[19] Green J, Thorogood N (2018). Qualitative methods for Health Research.

[20] Carvalho NO, Matos MFS, Belchior IFC, Araújo MB, Rocha CT, Neves BG (2021). Parents’ emotional and social experiences of caring a child with cleft lip and/or palate. Pesqui Bras Odontopediatria Clin Integr..

[21] Lindberg N, Berglund AL (2014). Mothers’ experiences of feeding babies born with cleft lip and palate. Scand J Caring Sci..

[22] Bradbury ET, Hewison J (1994). Early parental adjustment to visible congenital disfigurement. Child Care Health Dev..

[23] Grollemund B, Dissaux C, Gavelle P, Martínez CP, Mullaert J, Alfaiate T, et al. The impact of having a baby with cleft lip and palate on parents and on parent-baby relationship: the first French prospective multicentre study. BMC Pediatr. 2020;20:230. 10.1186/s12887-020-02118-5 PMC7236125 32423402

[24] Graham H (2004). Social determinants and their unequal distribution: clarifying policy understandings.

[25] Dahlgren G, Whitehead M (2021). The Dahlgren-Whitehead model of health determinants: 30 years on and still chasing rainbows. Public Health..

[26] Douthit NT, Alemu HK (2016). Social determinants of health: poverty, national infrastructure and investment. BMJ Case Rep..

[27] Hasanzadeh N, Khoda MO, Jahanbin A, Vatankhah M. Coping strategies and psychological distress among mothers of patients with nonsyndromic cleft lip and palate and the family impact of this disorder. J Craniofac Surg. 2014;25:441–5. 10.1097/SCS.0000000000000483 24481167

[28] Nidey N, Moreno Uribe LM, Marazita MM, Wehby GL (2016). Psychosocial well-being of parents of children with oral clefts. Child Care Health Dev..

[29] Divya K, Iyapparaja P, Raghavan A, Diwakar MP (2022). Accuracy of prenatal ultrasound scans for screening cleft lip and palate: a systematic review. J Med Ultrasound India..

